# Weakly supervised learning in thymoma histopathology classification: an interpretable approach

**DOI:** 10.3389/fmed.2024.1501875

**Published:** 2024-12-11

**Authors:** Chunbao Wang, Xianglong Du, Xiaoyu Yan, Xiali Teng, Xiaolin Wang, Zhe Yang, Hongyun Chang, Yangyang Fan, Caihong Ran, Jie Lian, Chen Li, Hansheng Li, Lei Cui, Yina Jiang

**Affiliations:** ^1^Department of Pathology, The First Affiliated Hospital of Xi'an Jiaotong University, Xi'an, Shaanxi, China; ^2^School of Computer Science and Technology, Xi'an Jiaotong University, Xi'an, Shaanxi, China; ^3^School of Information Science and Technology, Northwest University, Xi'an, Shaanxi, China; ^4^Department of Pathology, Ngari Prefecture People's Hospital, Ngari, Tibet, China

**Keywords:** thymoma, multi-instance learning, histopathology, interpretability, artificial intelligence

## Abstract

**Introduction:**

Thymoma classification is challenging due to its diverse morphology. Accurate classification is crucial for diagnosis, but current methods often struggle with complex tumor subtypes. This study presents an AI-assisted diagnostic model that combines weakly supervised learning with a divide-and-conquer multi-instance learning (MIL) approach to improve classification accuracy and interpretability.

**Methods:**

We applied the model to 222 thymoma slides, simplifying the five-class classification into binary and ternary steps. The model features an attention-based mechanism that generates heatmaps, enabling visual interpretation of decisions. These heatmaps align with clinically validated morphological differences between thymoma subtypes. Additionally, we embedded domain-specific pathological knowledge into the interpretability framework.

**Results:**

The model achieved a classification AUC of 0.9172. The generated heatmaps accurately reflected the morphological distinctions among thymoma subtypes, as confirmed by pathologists. The model's transparency allows pathologists to visually verify AI decisions, enhancing diagnostic reliability.

**Discussion:**

This model offers a significant advancement in thymoma classification, combining high accuracy with interpretability. By integrating weakly supervised learning, MIL, and attention mechanisms, it provides an interpretable AI framework that is applicable in clinical settings. The model reduces the diagnostic burden on pathologists and has the potential to improve patient outcomes by making AI tools more transparent and clinically relevant.

## 1 Introduction

Thymoma is a rare epithelial tumor originating from the thymus, and it is the most common thymic tumor. According to morphology, thymoma can be classified into five subtypes: A, AB, B1, B2, and B3 (1). Among these subtypes, types A and AB have a better prognosis, followed by type B1, while types B2 and B3 have a relatively poor prognosis (2).

In the diagnostic task of thymomas, pathologists are required to integrate the structural features at both the histological and cellular levels to make determinations. These features include histological characteristics such as lobular architecture and vascular clefts, as well as cellular characteristics. Notably, the primary focus in determining tumor type and biological behavior remains on the tumor cells themselves. For instance, in type A thymomas, tumor cell nuclei exhibit mild spindle or oval shapes, while type B thymomas demonstrate increasing nuclear pleomorphism (from mild irregularities to moderate), or increasing prominence of nucleoli, accompanied by a gradual enhancement in nuclear clustering (1).

However, there is currently a lack of specific molecular pathological markers for the classification of thymomas, which hinders the diagnosis and classification process (2). As a result, pathological diagnosis mainly relies on morphological features. Thymomas exhibit diversity in morphology and varying degrees of histological heterogeneity (3). The complexity of pathological morphology poses significant challenges for pathologists, often requiring further immunohistochemical staining and the integration of clinical presentations and imaging results to obtain a diagnosis. These processes are time-consuming and labor-intensive, leading to increased economic costs for patients.

The reproducibility of thymoma diagnosis among pathologists exhibits inadequacy, particularly concerning Type A, B3, AB, and B1/B2 types (4, 5). Incorrect interpretation of atypical lesions may lead to delays in necessary treatments (false negatives), and could potentially result in incorrect prognostic predictions for patients (6). This issue stems from multifaceted factors, encompassing discrepancies in experience and proficiency among pathologists, thereby fostering subjectivity and individual variances in classification (7). Furthermore, thymomas manifest diverse histological structures, notably AB or B1/B2 types, characterized by abundant lymphocytic infiltration amidst comparatively sparse tumor cell presence (8). Consequently, observing tumor cells becomes challenging when their count is low. Additionally, tumor heterogeneity frequently engenders the coexistence of multiple mixed subtypes within the same patient or even on the same slide (3). Pathologists may approach these mixed cases disparately, with some relying solely on one subtype for diagnostic adjudication (9). Currently, there is a lack of an effective method to assist pathologists in efficiently performing qualitative and quantitative analyses of these complex pathological morphologies.

In recent years, considerable advancements have been achieved in the domain of pathological diagnosis, primarily attributable to the advent of artificial intelligence (AI) (10). These strides encompass tumor cell detection and localization (11), assisted classification and grading, alongside the formulation of personalized treatment regimens and prognostic evaluations (12, 13). Leveraging the distinctive features of pathological images, AI has demonstrated remarkable efficacy in subtype identification across a spectrum of cancers, including lung (14), gastric (15), and colorectal cancers (16).

The current study (17) for thymoma classification used the slide and patch labels to classify the pathological information by fusing the 40×, 20×, and 10× scales with different self-attention and convolution modules. According to the result of classification, several transformer modules were used to realize the classification of thymoma. However, the study faces several issues that warrant consideration. Firstly, the annotation work is time-consuming and labor-intensive. The approach utilizes patch-level analysis, but detailed region annotation on the slides requires a significant amount of time and human resources. Secondly, the interpretability is insufficient. The current approach lacks adequate interpretability. Since the classification is based on patches, it is uncertain whether the model accurately captures cell features highly correlated with the classification.

**Figure 1:**
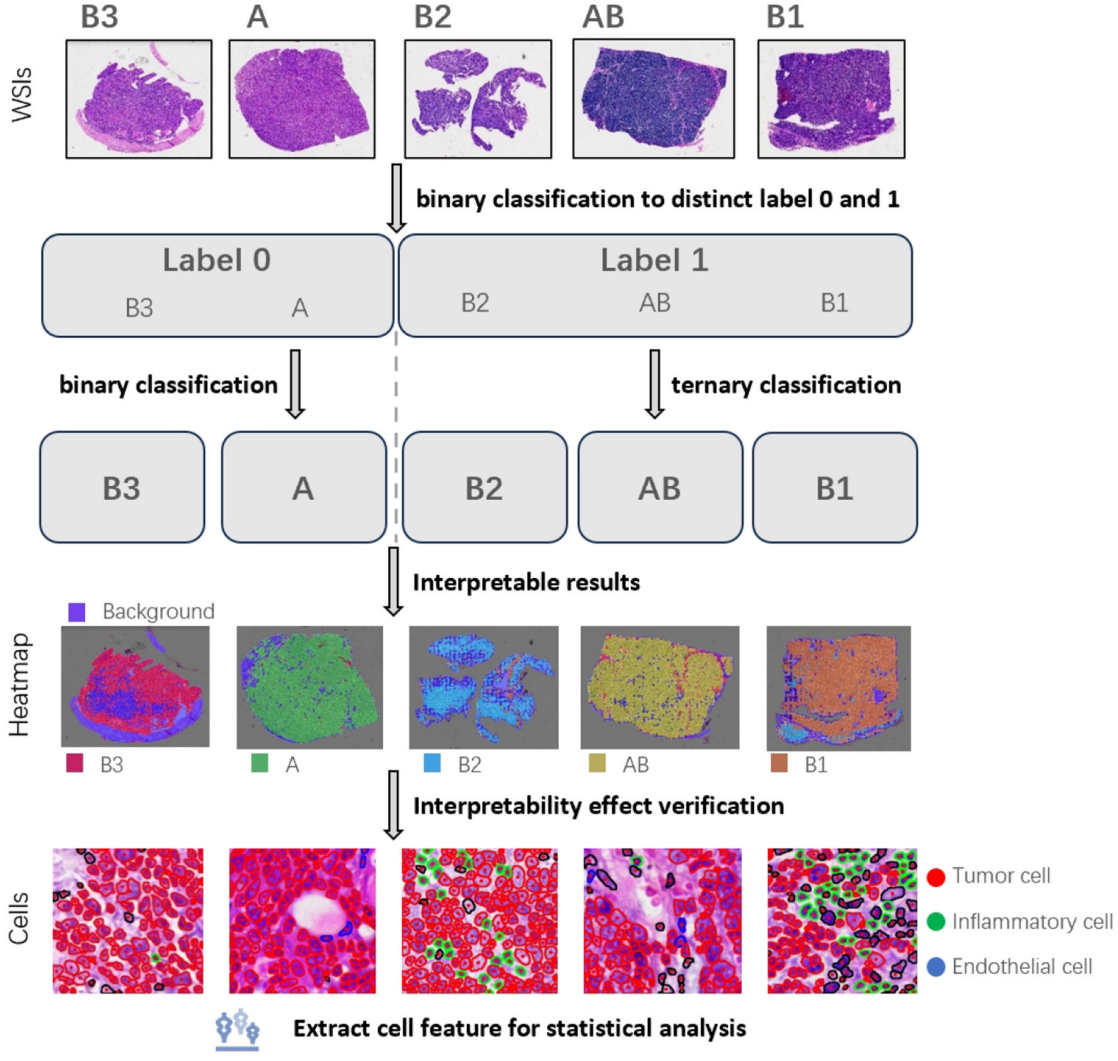
We used a divide-and-conquer approach to the five subtypes of thymoma. Firstly, the similar subtypes were classified into one category, and the classification with big difference was distinguished. Then these large categories are further subdivided, and finally achieve the detailed classification of five subtypes. Based on this, we plotted patch heatmap, and segmented and classified the cells. Next, we analyzed the characteristics of the cells in different patch categories to verify the correctness and interpretability of patch heatmap analysis.

To address the aforementioned challenges, we used only the slide-level labels for the multi-instance classification of the five subtypes of thymoma tumors. Given the high number of classes in a direct five-way classification task, achieving high model accuracy can be quite challenging. Therefore, we adopt a divide-and-conquer approach, which is shown in Figure 1. First, we group the morphologically similar subtypes (1, 18, 19), such as classifying A type and B3 type into one category, and the lymphocyte-rich AB type, B1 type, and B2 type into another category. This simplifies the five-class task into a binary classification problem. After completing this initial binary classification, we then perform separate binary and ternary classifications on the internal samples of each of the two main categories. This stepwise strategy allows us to ultimately achieve the full five-way classification of the thymoma subtypes. Concurrently, we used a multi-instance learning algorithm based on an attention mechanism, which employs weak supervision to classify the thymic tumor slides. By combining the divide-and-conquer strategy and the attention mechanism algorithm, we were able to improve the classification accuracy.

To demonstrate the interpretability of the classification model, we conducted visualizations and feature analyses. We used its attention scores to predict patch categories across the slide, generating a visualization heatmap. We then segmented cells by patch, extracted morphological features, and compared them to clinical standards. This revealed that there are significant differences in tumor cells within different histologic subtypes. These clinically-consistent findings validate the reliability and interpretability of our heatmap analysis, demonstrating the model's good interpretability.

This study highlights the value and significance of leveraging advanced AI techniques for the classification of thymoma subtypes. By employing a multi-instance learning algorithm and an attention mechanism, the study effectively addresses the challenges associated with the morphological diversity and histological heterogeneity of thymomas. The divide-and-conquer approach simplifies the complex five-way classification task, improving model accuracy and reliability. The validation of the heatmap analysis against clinical guidelines ensures the robustness and clinical relevance of the findings. These advances significantly benefit pathologists in improving diagnostic accuracy and efficiency. The model can be visualized for clinical interpretation, reducing the time needed for pathologists to locate key features, and is easily accepted by pathologists.

## 2 Materials and methods

**Figure 2:**
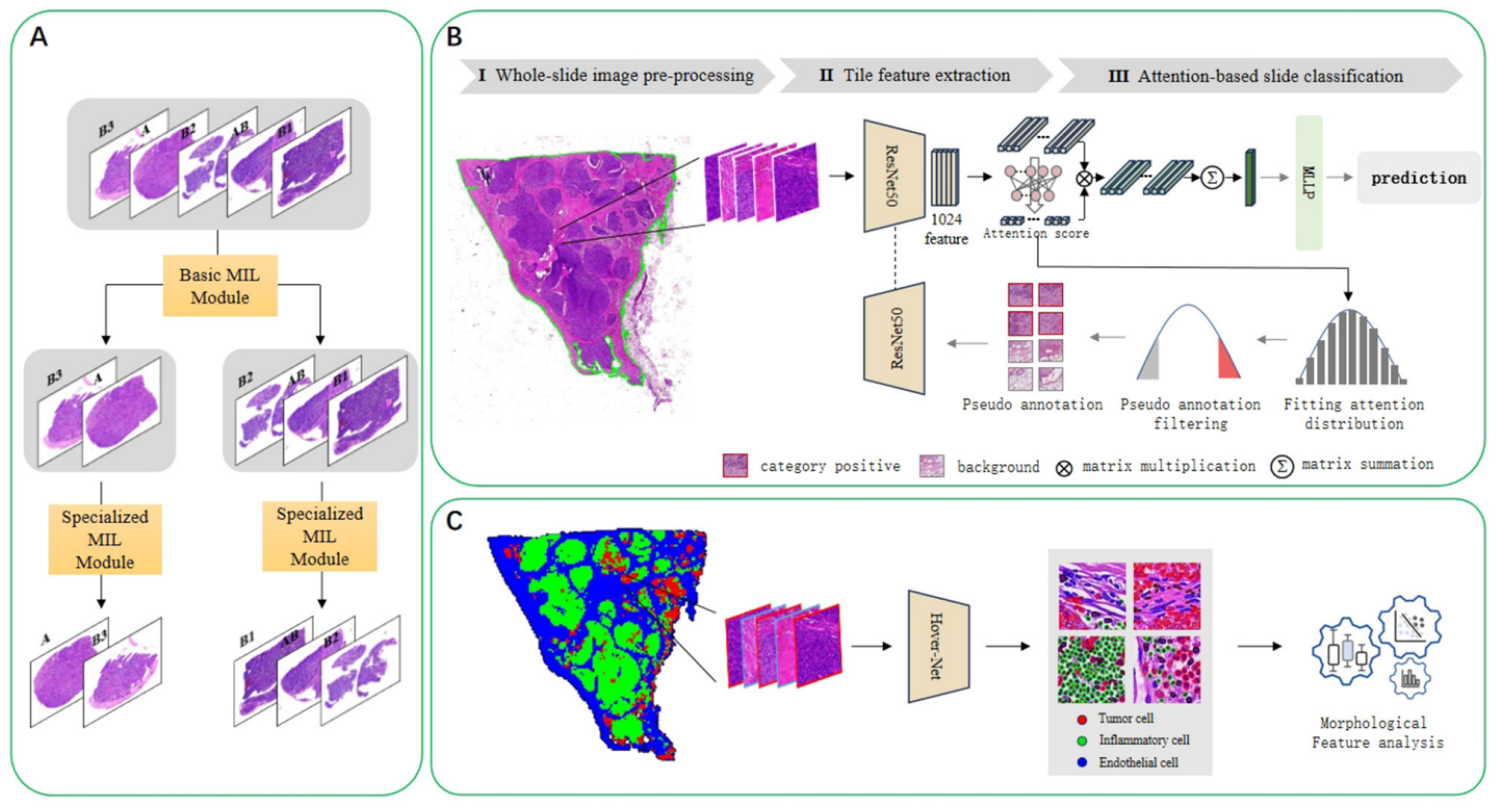
**(A)** Illustrates the implementation of the divide-and-conquer idea of the five-classification algorithm. Using the multi-instance learning method, the five classes are divided into two classes, and then further subdivided in the second step. **(B)** Presents the concrete implementation schematic diagram of the multi-instance learning algorithm. It consists of three steps: whole-slide image preprocessing, tile feature extraction, and attention-based slide classification. In addition to a simple iterative process, pseudo-label processing and continuous optimization of the feature extractor are also carried out. **(C)** Is a flowchart for analyzing cell characteristics. The HoverNet model is used to segment and classify cells, and then the characteristics of cells in different categories of slides are analyzed.

In this task, we have a collection of whole-slide images(WSIs) that belong to five categories (A, AB, B1, B2, and B3). Figure 2A is the overall flow chart. We adopt the idea of divide and conquer, first use HIPT method (20) to divide into two categories, and then use ABMIL (21) to internally subdivide these two categories to achieve five classifications. As shown in Figure 2B, there are three steps for multi-instance learning classification: whole-slide image preprocessing, tile feature extraction, and attention-based slide classification. In order to improve the performance of the model on thymoma data, an iterative strategy based on pseudo-labels was used to continuously optimize the feature extractor. Figure 2C shows the process of verifying the interpretability of the model. We draw heatmaps according to the type of patches, and analyse cell features within different kinds of the slides.

### 2.1 Dataset and pre-processiong

We analyzed 222 thymoma pathological slides and successfully classified them into five different categories. To ensure the accuracy of data annotation, we first removed all original labels from the slides. Then, three pathologists with different levels of experience independently diagnosed the slides. A category was confirmed when at least two pathologists agreed on the result; if all three opinions differed, a more experienced pathologist made the final determination. This process integrated views from pathologists with diverse expertise, effectively reducing individual biases and enhancing the accuracy and reliability of the diagnoses. According to this method, the slides were ultimately classified as Type A (21 cases), Type AB (83 cases), Type B1 (48 cases), Type B2 (49 cases), and Type B3 (21 cases).

To ensure the completeness and validity of our results, we rigorously divided the dataset into three distinct sets: training, validation, and testing. The training set included 141 slides for model training; the validation set consisted of 42 slides for verifying the model's performance after each training round; the testing set comprised 39 slides used to evaluate the model's ability to classify unseen data at the end of training. This segmentation strategy helped us more accurately assess the model's performance and ensured the reliability of our research results.

Additionally, we meticulously annotated 571 square patch images derived from effective tissue areas of the slides, each measuring 256 pixels in size. The annotation process began with an initial cell location identification using an open-source pre-trained HoverNet model, followed by a pathologist reviewing the results to ensure the accuracy of the annotations. This step not only enhanced the credibility of the data but also improved the subsequent model training outcomes.

Our study is approved by the Institutional Review Board (IRB) of the First Affiliated Hospital of Xi'an Jiaotong University (IRB Approval No. KYLLSL-2020-054). The IRB granted a formal waiver of informed consent for the use of these specimens. All collected data have been de-identified and do not contain any personal health information or identifiable labels. We, the authors, consent to the publication of this study and affirm that it meets the ethical standards required for publication.

### 2.2 Multiple instance learning

#### 2.2.1 Feature extraction

The feature extraction module is used to encode the extracted image patches as features. In the framework of multi-instance learning, the feature extraction module for pathological slide classification is usually decoupled from the feature aggregation module (22). This decoupling design primarily addresses memory constraints, as pathological slides often have high resolution, necessitating significant computational resources for feature extraction.

Therefore, separating the feature extraction module allows for more flexible control over memory usage. We consider the input slide *X* as a collection containing numerous instances and employ a convolutional neural network denoted as *E*(·) to extract features from individual small images. Representing the entire set of small images as *X* = {*x*_*i*}_*i* = 1^*N*^, with *x*_*i* signifying the i-th small image within the slide, the features encapsulated within the bag are represented as *Z* = {*z*_*i*}_*i* = 1^*N*^. Notably, *N* denotes the number of small images within the slide, while the size of the tissue in the slide remains uncertain, thus constituting a variable value.

Additionally, the initialization of weights in the feature extraction module is an important consideration. Some studies adopt weights pre-trained on large-scale datasets such as ImageNet (23–25), while others opt for self-supervised learning techniques to initialize weights (20, 26). Due to the substantial computational resources required for using self-supervised weights, in this experiment, we utilized weights pre-trained on ImageNet for the ResNet50 network. Considering the significant differences between natural images and pathological images, we retrained the feature extraction model using pseudo-labeling, which will be detailed in Section 2.2.3.

#### 2.2.2 Feature aggregation

The role of the feature aggregation module is to obtain the overall feature representation of the entire bag by aggregating the instance features obtained from the feature extraction. In the slide classification tasks, each slide has a known label *Y* ∈ {1, ..., *C*}, where *C* represents the number of classes. Additionally, each patch within a slide has an unknown label *y* ∈ {1, ..., *C, C*+1}, where *C*+1 represents the background class (e.g., non-tumoral regions). The primary objective of multiple instance learning lies in fitting a function *M*(·) utilizing the instance feature *Z* of the entire bag as input to predict the label *Y* of the bag. Conventionally, the approach entails learning a feature aggregation function denoted as *F*(·), which aggregates the features *O* from all instance feature sets *Z*. Subsequently, a classification function *C*(·) is utilized to derive the final predicted category *Y*′. Formally, the process is defined as follows:


(1) $$\begin{array}{l} M=C\left(F\left(E\left(X\right)\right)\right). \end{array}$$

This paper utilizes an attention-based feature aggregation module, formalized as follows:


(2) $$F=\sum_{i=1}^{N}a_{i}z_{i},$$

*a*_*i*_ is a learnable weight for *z*_*i*_. In this module, attention scores are assigned to all patch features obtained through feature extraction for each category. Subsequently, the attention scores are multiplied by the weighted features and summed to obtain the feature representation of the bag for each category.

#### 2.2.3 Pseudo-label assignment

Pseudo-labeling is used to retrain the feature extraction model. Within the original multiple instance learning framework, we utilized an auxiliary ResNet50 model with the same structure for image patch classification, sharing weights with the original feature extraction network. Initially, the image patches are unlabeled. We selected a subset of the image patches and assigned pseudo-labels to them. These pseudo-labeled image patches are then used to train the feature extraction model.

For the selection of patches, we initially assign a confidence score to each patches. Given a patch *x*_*n*_, a confidence score *s*_*n*_ is assigned to each patch following the rules below:


(3) $$\begin{array}{l} s_{n}=a_{n}^{c_{i}}\cdot p_{n}^{c_{i}}, \end{array}$$

where *c*_*i*_ is the *i*-th category of the patch, *c*_*i*_ ∈ {0, 1, ..., *C*}. $a_{n}^{c_{i}}$ represents the attention score of patch *x*_*n*_ for category *c*_*i*_, obtained through the feature aggregation part of the current best model's multi-instance learning classification head. $p_{n}^{c_{i}}$ denotes the category score of patch *x*_*n*_ for category *c*_*i*_, which is the classification score for category *c*_*i*_ on the current best patch classifier. Then, we select patches suitable for optimizing the feature extractor and assign pseudo-labels to them. We sort the set of patches based on the confidence values corresponding to the slide's categories from highest to lowest, select the top *k* patches with confidence scores, and label the category of these patches as the corresponding slide category. We then select the bottom *k* patches with confidence scores and label the category of these patches as the background category *C*+1.

### 2.3 Interpretability verification

First, we draw visual heatmaps for every slide. Traditional multiple instance learning methods use the distribution of attention scores to generate a visualization heatmap distribution of image patches, but they can only display binary results on the original image. We use the prediction results of an auxiliary classifier to generate the heatmap. The predicted patch categories use the ResNet50 module in Figure 2B. All the patches in a single slide can be combined to generate a heatmap, as shown in Figure 4. Different category results correspond to different colors. There may be multiple types of patches in a slide. To demonstrate the interpretability of the heatmap, we used a HoverNet model (27) to segment cells in all patches within the original slides and classify them. And we extracted the morphological features of the cells and statistically analyzed.

The HoverNet model is trained and tested by the annotated images. We apply the trained model to segment all patches in the entire set of WSIs. Based on the segmentation results, we calculate various features for each patch, categorized by different classes, including tumor cell pixel size, major and minor axis lengths, elongation ratio, solidity, mean curvature, and color intensity, and so on. Additionally, we also compute 12 cell features in each slide like the proportions and average sizes of tumor cells and inflammatory cells.

Based on the analysis of different cellular characteristic data, we counted the cell features across all slides according to the various thymoma subtypes, followed by statistical analysis. Our data includes five types of tissue subtypes and two types of cell types, totaling ten groups. Among the 10 groups, we selected five tumor cell groups for comparison and calculated the differences between these five groups. When calculating the differences between groups, we first performed the Kruskal–Wallis test, obtaining the chi-squared value, degrees of freedom (df), and *p*-value. The chi-squared value serves as the test statistic for the Kruskal–Wallis test, measuring the degree of median differences among groups. A larger chi-squared value indicates more significant inter-group differences. The degrees of freedom are calculated using the formula: number of groups −1. The *p*-value indicates the probability of observing the results or more extreme results under the null hypothesis. If the *p*-value is < 0.05, it usually means that the original assumption (that the medians are equal in each group) is rejected and indicates that at least one group has a significantly different median.

To further identify between-group differences, we performed pairwise comparisons of these five sets of data and applied Dunn's test as a *post hoc* test after the Kruskal–wallis test to determine which specific group pairs showed significant differences. For each pair, we computed the *Z* statistic (*Z*-value), the unadjusted *p*-value (*p*.unadj), and the adjusted *p*-value (*p*.adj). The *Z*-value reflects the degree of difference between the two groups, with larger absolute values (whether positive or negative) indicating more significant intergroup differences. A positive *Z*-value indicates that the median of the first group is higher than that of the second group, while a negative *Z*-value indicates the opposite. *p*.unadj is used to assess the significance of differences between groups, with smaller *p*-values indicating significant differences. *p*.adj is the *p*-value adjusted for multiple comparisons, controlling the false discovery rate using the Benjamini-Hochberg method. We set the cut-off value at 0.05, so if *p*.adj is <0.05, it can be concluded that there is a significant median difference between the two groups. The significance grading criteria are as follows: *p*-values <0.05 are marked with ^*^, <0.01 with ^**^, <0.001 with ^***^, and <0.0001 with ^****^.

## 3 Results

For the experiments, the Adam optimizer is chosen. The multi-instance learning classifier is trained using a batch size of 1, an initial learning rate of 1*10^−4^, and a decay factor of 0.5 every 10 epochs. Meanwhile, the slide block classifier is trained with a batch size of 64, an initial learning rate of 5*10^−5^, and a decay factor of 0.5 every 10 epochs.

### 3.1 Experiment results

Three methods is utilized to analyze the experimental results, comprehensively evaluating the algorithm's performance and providing a basis for future work. First, we employed confusion matrices and ROC curves to assess the accuracy and precision of the classification results. Meanwhile, we plotted interpretability heatmap to visually demonstrate the distribution of patch categories within each slide. Furthermore, we adopted statistical analysis to calculate the differences in characteristics of cells from different categories, and visualized these differences using violin plots.

#### 3.1.1 Classification result

**Figure 3:**
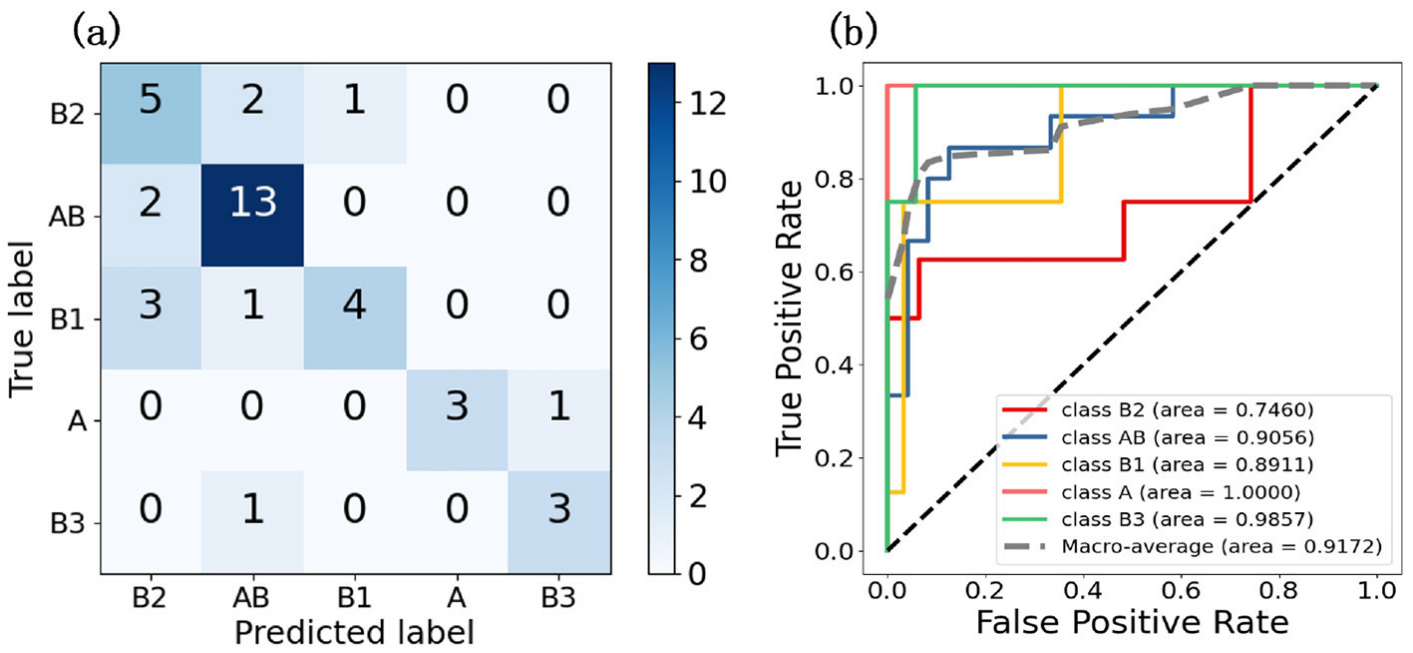
These two graphs are the result graphs of five classifications, **(A)** is the confusion matrix and **(B)** is the ROC curve.

To evaluate the model's performance on five thymic tumor subtypes, we generated confusion matrices and ROC curves. In Figure 3A, the confusion matrix shows the five-class classification accuracy is 0.7179, indicating the model can effectively distinguish the five thymic tumor subtypes. The ROC curve analysis further reveals strong discriminative power, with a macro-average AUC of 0.9425 in Figure 3B. This high AUC value demonstrates the model's ability to accurately classify the different thymic tumor subtypes. The high accuracy and AUC values indicate the model is well-suited for reliable subtype differentiation, which is helpful for guiding personalized treatment strategies for thymic tumor patients.

To further demonstrate the effectiveness of pseudo-label assignment, we conducted an ablation experiment. Under the strategy of divide and conquer, the model achieved a five-class accuracy of only 0.55 and an AUC of 0.8609, thereby demonstrating the effectiveness of the pseudo-labeling strategy. The divide and conquer strategy enabled the model to acquire preliminary class discrimination capabilities. Pseudo-labels, derived from the model's existing discriminative information—specifically the attention distribution—further optimized the feature extraction network. This optimization allowed the model to learn the typical features distinguishing different classes, thereby enhancing the overall classification performance.

#### 3.1.2 Interpretability result

**Figure 4:**
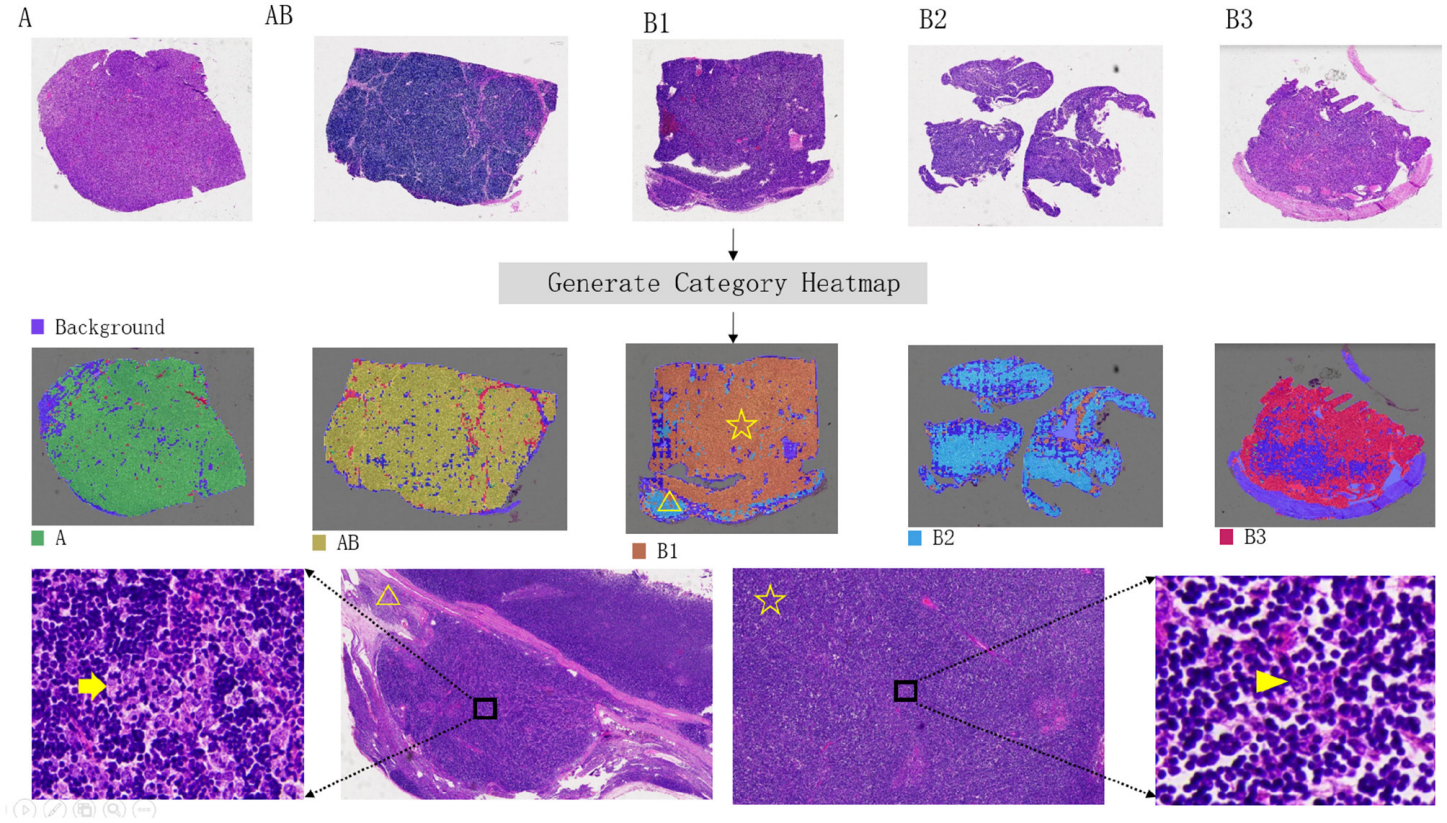
Visual heatmap of five thymoma subtypes. The top panel presents hematoxylin and eosin (H&E) stained overview images of each thymoma subtype. The middle panel provides corresponding visualization heatmaps, highlighting the distinctive features of each subtype. In the bottom panel, an example of a B1-type thymoma is shown, with the heatmap indicating a B2-type signal in the lower-left corner (yellow triangle). Further review of H&E sections in this area confirmed a small focus of B2-type thymoma. The yellow arrow in the H&E image marks clustered tumor cells, characteristic of B2 subtype, in contrast to the scattered, single-cell distribution typical of the B1 subtype (indicated by the arrowhead).

The interpretability heatmap is shown in Figure 4. Different color areas correspond to different patch categories, and the patch distribution in a heatmap of different categories is different. It is worth mentioning that there is more than one type of patch in a slide, as in a heatmap with a slide class, there are also patches predicted to be B3 classes. To verify the interpretability of the model heatmap, we conducted a follow-up analysis.

**Figure 5:**
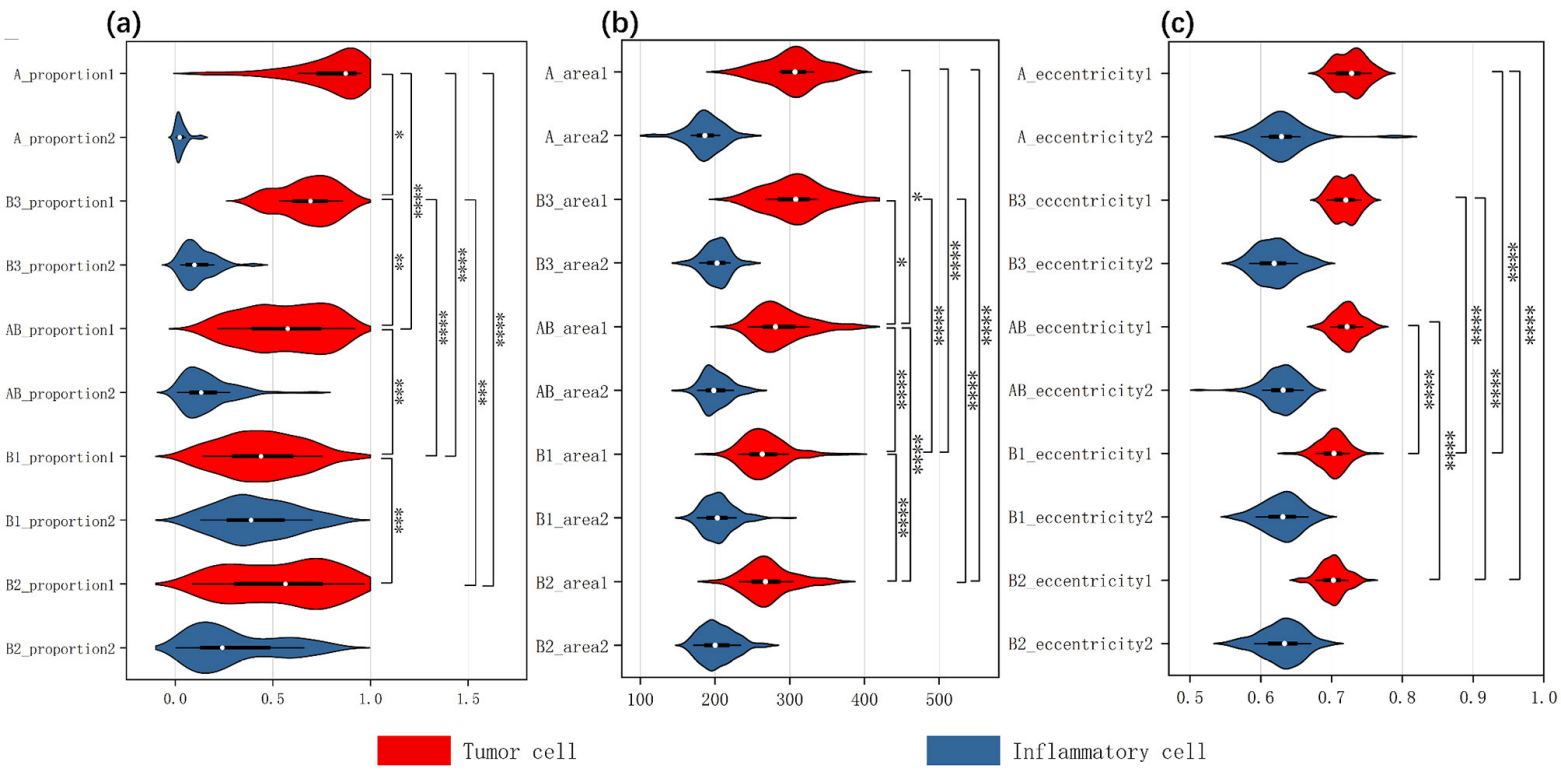
Three inverted violin plots show the data distribution of thymic tumor cells across three characteristics: **(A)** the proportion of two cell types, **(B)** cell area size, **(C)** cell eccentricity. The white dot represents the median value of the data, while the width of each violin indicates the density of the data points. Wider sections correspond to a higher density of observations and the length reflects the overall distribution of the data across each group. The red violin plots represent tumor cells, while the blue ones represent inflammatory cells. We analyzed the two cellular characteristics within five subtypes of thymic tumors, with each plot containing data from ten groups. We assessed intergroup differences among tumor cells, using an asterisk (*) to denote significant differences; if two groups are connected by an asterisk, it indicates a significant difference in the distribution of tumor cells within those tissue types.

We employed the HoverNet model to segment and classify the cells within each patch. We trained the model using the annotated patches, allowing it to provide precise cell coordinates and classifications. We then applied the trained HoverNet model to segment and classify the cells in all original patches. From this, we extracted 12 features for both tumor cells and inflammatory cells, and generated violin plots to analyze the significant differences. As shown in Figure 5, the comparison results reveal distinct differences in the characteristics of inflammatory cells vs. tumor cells across the different slides. This detailed analysis sheds light on the unique signatures of these cell types, which is crucial for accurate subtype identification. Additional comparative analysis of other cell features is shown in the Appendix.

To ensure the reliability of the subsequent cell analysis, we calculated relevant metrics for both the segmentation and classification aspects of the HoverNet model. The effects are shown in Tables 1 and 2. For segmentation, the Dice coefficient was 0.9393, indicating strong similarity between the segmentation and ground truth. The FAST AJI score was 0.7694, with an improved FAST AJI+ of 0.8044. The DQ and SQ metrics were 0.8832 and 0.8906 respectively, yielding a DQ*SQ of 0.7880. Turning to classification, the overall F1 score was 0.9136 and the accuracy was 0.7529. The Micro-average F1 score was 0.8152, with 0.8427 for tumor cells and 0.7388 for inflammatory cells. These excellent segmentation and classification metrics confirm the HoverNet model's robust performance, providing a reliable foundation for the subsequent cell-level analysis and comparison across different slides.

**Table 1:** Testing metrics for segmentation by HoverNet.

**Metrics**	**Value**
Dice coefficient	0.9393
Fast AJI	0.7694
Fast AJI+	0.8044
DQ	0.8832
SQ	0.8906
DQ*SQ	0.7880

**Table 2:** Testing metrics for classification by HoverNet.

**F1 score**	**Value**
Micro-average F1 score	0.8152
Tumor cell	0.8427
Inflammatory cell	0.7388

After obtaining stable cellular characteristics, we analyzed the differences between the cells by selecting three key features: Cell Proportion, which refers to the ratio of the total number of a specific cell type in a given slide to the total number of cells across all 222 slides; Cell Area, indicating the average size of that cell type; and Eccentricity, defined as the ratio of the length of the long axis to the length of the short axis of the cell. We assessed the variations among tumor cells across different tissue subtypes using the Kruskal–Wallis test, which yielded significant results for all three characteristics: for Cell Proportion, chi-squared = 89.66 (df = 4, *p*-value < 2.2*E*−16); for Cell Area, chi-squared = 93.17 (df = 4, *p*-value < 2.2*E*−16); and for Eccentricity, chi-squared = 140.76 (df = 4, *p*-value < 2.2*E*−16). All *p*-values were < 0.05, indicating significant differences in the medians among the different groups. The specific results of the pairwise *post hoc* tests are presented in the Appendix.

Additionally, we created violin plots to visually represent the data for each group. Figures 5A–C display the characteristics of cell proportion, area size, and eccentricity, respectively, effectively illustrating the distribution and median of each group. Each plot contains data from ten groups, with each group represented by an inverted violin shape. The white dot indicates the median of the data for that group, while the width of the violin reflects the density of the data, and the length represents the overall distribution. On the right side of each plot, we present the significance of differences in tumor cells among the various thymoma subtypes, indicating that significant differences exist between the different slide subtypes.

The result indicates that the observed differences in cell proportions, area and eccentricity align with known characteristics and pathological features of each thymoma subtype. This consistency underscores the accuracy of our explainable results and the validity of the analysis.

### 3.2 Comparative test

In order to verify the effectiveness of the proposed method, we conducted a comparative experiment of four machine learning methods and three multi-instance methods without the divide-and-conquer idea. And the calculation formula for various performance metrics is as follows:


(4) $$\begin{array}{l} Acc=\frac{TP+TN}{TP+FP+TN+FN}, \end{array}$$

(5) $$\begin{array}{l} Precision=\frac{TP}{TP+FP}, \end{array}$$

(6) $$\begin{array}{l} Recall=\frac{TP}{TP+FN}, \end{array}$$

(7) $$\begin{array}{l} F1=2*\frac{Precision*Recall}{Precision+Recall}. \end{array}$$

Four machine learning methods specifically refer to Random Forest, Logistic Regression, Support Vector Machine (SVM), and K-Nearest Neighbors (KNN). Due to the large size of the WSI, it is more expensive to directly use the full-slide image as input for machine learning training. Therefore, we utilized the information extracted from HoverNet for tumor cells, inflammatory cells, and endothelial cells in machine learning training. Specifically, we used the size, number, density, and row spacing of these cells as characteristic parameters in our machine learning classification experiments.

The outcome metrics for each method are shown in Table 3, and all methods work poorly except the method in this article. Logistic Regression is sensitive to outliers, and its accuracy of 0.5507. When faced with high levels of noise in the dataset, Support Vector Machines may be prone to overfitting, and its accuracy is 0.5797. In high-dimensional datasets, K-Nearest Neighbors may encounter the curse of dimensionality, which can impact its accuracy (0.5942). Random Forest's accuracy remains at 0.6667, which demonstrates robustness to missing data and outliers. The three multi-instance learning methods that do not use the divide-and-conquer approach, HIPT, ABMIL, and TransMIL, have respective accuracies of 0.6000, 0.4500, and 0.5000.

**Table 3:** Performance comparison of different machine learning methods for thymoma prediction.

**Algorithm**	**Accuracy**	**Precision**	**Recall**	**F1 score**
Random forest	0.6667	0.7299	0.6545	0.6630
Logistic regression	0.5507	0.5743	0.5881	0.5463
SVM	0.5797	0.5587	0.5324	0.5395
KNN	0.5942	0.4831	0.4910	0.4828
HIPT	0.6000	0.6627	0.5856	0.6027
ABMIL	0.4500	0.4750	0.3439	0.4115
TransMIL	0.5000	0.6167	0.4439	0.4496
Ours without pseudo-label	0.5500	0.5373	0.5556	0.5243
Ours	0.7179	0.7403	0.7179	0.7175

The results of four machine Learning methods are less effective, most likely due to the heterogeneity of thymoma histological patches, a slide may contain regions belonging to multiple different categories, which results in extracted input information that does not belong to a single category. As a result, it becomes challenging to effectively classify the slides based on cell information at the slide level. At the same time, using HIPT, ABMIL and TransMIL Multi-Instance Learning methods to predict the five-classification, the effect is not ideal. This is mainly because there are many different types of thymoma to be classified, but only 222 WSI can be used for training. However, this paper adopts the idea of divide-and-conquer, first divides the samples into two categories with high similarity, then subdivides the two categories further. The strategy of hierarchical classification makes the final prediction more accurate, with an accuracy of 0.7179.

## 4 Discussion

In this study, we introduce a novel approach to thymoma histopathology classification that leverages weakly supervised learning combined with a divide-and-conquer strategy. Unlike previous studies, our method only requires slide-level labels, significantly reducing the need for detailed annotations. Additionally, we have incorporated visualization techniques that provide interpretable results, which have been clinically validated to ensure their reliability. Our approach not only demonstrates improved classification performance but also offers a potential framework that could inspire further research in the classification of various types of tumors.

We implemented a weakly supervised learning approach integrated with a divide-and-conquer strategy, achieving significant classification performance using only slide-level labels. Traditional studies in this field require detailed patch-level annotations (17), which are labor-intensive and time-consuming. The adoption of the multiple instance learning (MIL) method (28) is motivated by the high resolution of whole slide images (WSI) and inherent label uncertainty. MIL effectively addresses the challenges of large-scale high-resolution image data and achieves reliable predictions with weak supervision. This pseudo-label-based MIL method leverages both bag-level and instance-level information, enhancing classification performance and robustness. Additionally, the use of pseudo-labels optimizes feature extraction weights, improving the recognition of critical instances. The divide-and-conquer strategy has been applied successfully in numerous heterogeneous tumor segmentation tasks, such as gliomas (29) and hepatic lesions (30). Experimental results have demonstrated varying degrees of performance improvement across these tasks. Specifically, our experimental results show that this strategy significantly enhanced detection accuracy from 0.4500 to 0.7179. This approach has proven effective in overcoming the challenges posed by limited data and multiple tumor categories.

Our visualization technique distinguishes itself by displaying multiple tumor categories within a single slide, which is especially valuable in the context of thymoma due to its inherent heterogeneity. Thymomas often exhibit considerable histopathological diversity, making accurate diagnosis challenging and leading to potential inconsistencies among pathologists (3). Previous approaches (23) have typically employed attention score heatmaps to highlight areas associated with a single classification output, limiting the ability to convey the presence of coexisting tumor types within the same image. Our method, in contrast, provides a more nuanced and comprehensive visualization by simultaneously depicting multiple tumor categories, allowing pathologists to recognize and appreciate the heterogeneity within the sample more effectively. This dual approach not only enhances understanding of the overall tumor landscape but also reduces potential diagnostic variability by providing pathologists with a tool to observe diverse tumor characteristics in a single view. Furthermore, the multi-instance learning model offers explanatory heatmaps that go beyond simple classification, offering insights that are crucial in clinical decision-making and in managing the diagnostic complexities of thymoma.

To ensure the reliability of our visualizations, we conducted a detailed clinical validation analysis. This involved measuring the characteristics of different cell types and comparing them against histological definitions of thymoma subtypes. Our results confirmed that our measurements align with the established morphological criteria, demonstrating fewer lymphocytes in A and B3 types compared to other subtypes. Additionally, distinct differences in tumor cell nuclei sizes and shapes between A and B3 types and B1 and B2 types were observed, consistent with pathologists' observations. Despite similar median sizes of tumor cells in A and B3 types, the size of the nuclei varied significantly, indicating greater nuclear atypia in B3 thymomas. For the differentiation diagnosis between morphologically similar AB type and B1, B2 types, our results show significant differences in tumor cell area and curvature, which align with the intrinsic characteristics of AB type thymoma tumor cells being distinct from B type tumor cells. This nuanced understanding underscores the importance of algorithmic interpretability in refining diagnostic criteria and improving the accuracy of pathological assessments. Our findings demonstrate that interpretable models foster greater trust and facilitate the identification and mitigation of biases, enhancing the robustness and fairness of image-based predictive systems (31).

The human-slide interaction during slide examination allows for detailed presentations, such as highlighting the thoroughly examined focuses that contributed to reaching the precise diagnosis along with the used magnification levels. This kind of highlighting could help trainees find initial orientation when starting to look for abnormalitiess (32). In the real clinical pathological diagnosis process, professionals can quickly extract overarching features from an image and construct a nuanced perceptual representation that swiftly establishes the connection between visual stimuli and a diagnosis. This aptitude is likely bolstered by memory patterns formed through prior experiences, which are activated upon encountering the stimulus. Such a process requires the accumulation of diagnostic experience and consumes a considerable amount of time (6). Multi-class visualization has the potential to assist pathologists in reducing the time needed to locate key diagnostic areas, thereby enhancing diagnostic efficiency.

There are several limitations to our study that warrant further investigation. First, our dataset is derived from a single-center, which may limit the generalizability of our findings. Future research should include multi-center data to validate and enhance the robustness of our results. Such an approach would provide a broader perspective and ensure that the findings are applicable across different populations and clinical settings. Second, the clinical significance of classifying thymoma subtypes, especially those with mixed subtype components, is not fully understood. This limitation restricts our ability to interpret the behavior and treatment responses of these mixed subtypes. Exploring the implications of these classifications is crucial, as it can offer valuable insights into prognosis and therapy optimization. Third, our study does not address the diagnostic efficacy of artificial intelligence (AI) models in comparison to pathologists with varying levels of experience. This gap in the research prevents a comprehensive assessment of how AI can be effectively integrated into clinical workflows. Comparing the performance of AI models with both novice and expert pathologists is essential to determine their potential to enhance diagnostic accuracy and efficiency in diverse clinical environments.

Our future work will further evaluate the impact of visualization on improving the diagnostic efficiency of pathologists and explore optimal human-computer interaction modes to enhance working efficiency for medical professionals.

## 5 Conclusions

In response to the challenges in classifying thymoma subtypes, we employed a divide-and-conquer strategy and an attention-based multi-instance learning algorithm, achieving high accuracy in limited data volume. Based on our work and medical knowledge, we analyzed tumor cell characteristics to explore the model's interpretability in depth. However, the clinical significance of mixed subtype classifications remains unclear. Additionally, the diagnostic efficacy of AI models compared to pathologists with varying experience levels needs further investigation. Future research should include more multi-center data, explore mixed subtype implications, and compare AI performance with pathologists to enhance clinical integration.

## Data Availability

The raw data supporting the conclusions of this article will be made available by the authors, without undue reservation.
