# Supplementary material for: Weakly supervised learning in thymoma histopathology classification: an interpretable approach
Source: Front Med (Lausanne). 2024 Dec 11;11:1501875. doi: 10.3389/fmed.2024.1501875 (PMC11668976; doi:10.3389/fmed.2024.1501875)
Supplement: Supplementary file 1 [file Table_1.docx]

| Comparison | Z-value | P.unadj | P.adj |
| --- | --- | --- | --- |
| A proportion1 - AB proportion1 | 5.7074 | 1.15E-08 | 2.87E-08 |
| A proportion1 - B1 proportion1 | 8.3917 | 4.79E-17 | 4.79E-16 |
| AB proportion1 - B1 proportion1 | 4.3747 | 1.22E-05 | 2.43E-05 |
| A proportion1 - B2 proportion1 | 6.1634 | 7.12E-10 | 3.56E-09 |
| AB proportion1 - B2 proportion1 | 0.7522 | 4.52E-01 | 4.52E-01 |
| B1 proportion1 - B2 proportion1 | -3.6079 | 5.14E-04 | 3.09E-04 |
| A proportion1 - B3 proportion1 | 2.0883 | 3.68E-02 | 4.09E-02 |
| AB proportion1 - B3 proportion1 | -3.0921 | 1.99E-03 | 2.48E-03 |
| B1 proportion1 - B3 proportion1 | -5.8001 | 6.63E-09 | 2.21E-08 |
| B2 proportion1 - B3 proportion1 | -3.5554 | 3.77E-04 | 5.39E-04 |

TABLE 1 Table of Post Hoc Test Results for Tumor Cell Proportion Characteristics
