# Supplementary material for: Weakly supervised learning in thymoma histopathology classification: an interpretable approach
Source: Front Med (Lausanne). 2024 Dec 11;11:1501875. doi: 10.3389/fmed.2024.1501875 (PMC11668976; doi:10.3389/fmed.2024.1501875)
Supplement: Supplementary file 2 [file Table_2.docx]

| Comparison | Z-value | P.unadj | P.adj |
| --- | --- | --- | --- |
| A area1 - AB area1 | 2.4567 | 1.40E-02 | 1.75E-02 |
| A area1 - B1 area1 | 6.3758 | 1.82E-10 | 6.07E-10 |
| AB area1 - B1 area1 | 6.3970 | 1.58E-10 | 7.92E-10 |
| A area1 - B2 area1 | 5.3619 | 8.23E-08 | 1.65E-07 |
| AB area1 - B2 area1 | 4.7329 | 2.21E-06 | 3.69E-06 |
| B1 area1 - B2 area1 | -1.6326 | 1.03E-01 | 1.14E-01 |
| A area1 - B3 area1 | -0.0579 | 9.54E-01 | 9.54E-01 |
| AB area1 - B3 area1 | -2.5549 | 1.06E-02 | 1.52E-02 |
| B1 area1 - B3 area1 | -6.5126 | 7.39E-11 | 7.39E-10 |
| B2 area1 - B3 area1 | -5.4884 | 4.05E-08 | 1.01E-07 |

TABLE 2 Table of Post Hoc Test Results for Tumor Cell Area Characteristics
