# Supplementary material for: Weakly supervised learning in thymoma histopathology classification: an interpretable approach
Source: Front Med (Lausanne). 2024 Dec 11;11:1501875. doi: 10.3389/fmed.2024.1501875 (PMC11668976; doi:10.3389/fmed.2024.1501875)
Supplement: Supplementary file 3 [file Table_3.docx]

| Comparison | Z-value | P.unadj | P.adj |
| --- | --- | --- | --- |
| A eccentricity1 - AB eccentricity1 | 1.1517 | 2.49E-01 | 3.12E-01 |
| A eccentricity1 - B1 eccentricity1 | 6.5695 | 5.05E-11 | 1.26E-10 |
| AB eccentricity1 - B1 eccentricity1 | 8.8472 | 8.98E-19 | 4.49E-18 |
| A eccentricity1 - B2 eccentricity1 | 6.7167 | 1.86E-11 | 6.20E-11 |
| AB eccentricity1 - B2 eccentricity1 | 9.0595 | 1.31E-19 | 1.31E-18 |
| B1 eccentricity1 - B2 eccentricity1 | 0.2629 | 7.93E-01 | 7.93E-01 |
| A eccentricity1 - B3 eccentricity1 | 1.5520 | 1.21E-01 | 1.72E-01 |
| AB eccentricity1 - B3 eccentricity1 | 0.8222 | 4.11E-01 | 4.57E-01 |
| B1 eccentricity1 - B3 eccentricity1 - | 4.6468 | 3.37E-06 | 5.62E-06 |

TABLE 3 Table of Post Hoc Test Results for Tumor Cell Eccentricity Characteristics
